# Enrolling the body as active agent in cancer treatment: Tracing immunotherapy metaphors and materialities

**DOI:** 10.1177/03063127231199217

**Published:** 2023-09-27

**Authors:** Julia Swallow

**Affiliations:** Centre for Biomedicine, Self and Society, Old Medical School, Edinburgh, Scotland, UK

**Keywords:** immunotherapy, cancer, metaphor, embodied experience, materiality

## Abstract

Immunotherapy is heralded as the ‘fifth pillar’ of cancer therapy, after surgery, radiotherapy, chemotherapy and genomic medicine. It involves ‘harnessing’ patients’ own immune system T-cells to treat cancer. In this article, I draw on qualitative interviews with practitioners working in oncology and patients in the UK, to trace metaphorical and discursive framing around immunotherapy. Immunotherapy aims to restore the functioning of the immune system to detect cancer (non-self), working with the self/non-self model that pervades immunology discourse more widely. Practitioners draw on metaphors that cement this self/non-self model, and that tend to depict the relationship between cancer and the immune system as an internal battle. Yet the discursive framing around immunotherapy also involves shifts that emphasize the body’s own capacity to heal, where it is framed as ‘gentle’ or ‘tolerable’ on the body. Through this discursive shift, immunotherapy refigures the antagonism associated with the self/non-self model in the context of cancer. Analysing patients’ embodied experiences of treatment, this article attends to the material realities and tensions provoked by this shift in discursive framing. This article contributes to feminist STS analyses of immunology discourse, and extends this literature by arguing that it is critical to address the material stakes of these discursive shifts by paying attention to patients’ day-to-day experiences of treatment. The discursive framing of immunotherapy brings into being new forms of embodied patienthood in the context of cancer.

There is scarcely any better example of the ways in which culture and science are bound up together than the well-told story of military metaphors in the discipline of immunology.

–A. Martin (2010, p. 25)

Immunotherapy is the latest in a series of developments in the treatment of cancer, heralded as the ‘fifth pillar’ of cancer therapy, after surgery, radiotherapy, chemotherapy and personalized genomic medicine (Harris, 2018; Finck et al., 2020). Immunotherapy aims to restore the functioning of the immune system to recognize cancer. In so doing, positioning the patient’s own body as an active agent in disease treatment, with the potential to increase survival time via long-term management of cancer, or possibly even its cure (Canavan, 2017). At the same time, there are uncertainties in predicting individual response rates, managing long-term side effects and predicting prognosis (*Lancet*, 2018). The treatments are also highly toxic: Enrolling or activating the immune system to target cancer can lead to inflammation in various organs of the body, with patients developing long-term chronic conditions such as pancreatitis and colitis (*Lancet*, 2018).

Over the past ten years in the UK, two types of immunotherapy treatments have emerged in clinical practice and/or are currently being tested as part of experimental clinical trials. The first type includes checkpoint inhibitors to treat, for example, melanoma, renal, and some lung cancers. Cancers make high levels of proteins that suppress T-cells (the mechanisms that recognize or kill cancer cells) and checkpoint inhibitors ‘block proteins that stop the immune system from attacking the cancer cells’ (Cancer Research UK, 2021). The second type of immunotherapy includes adoptive cell transfer therapies such as chimeric antigen receptor T (CAR T)-cell therapy, which involves reprogramming the patient’s own T-cells to be administered to the patient to target their cancer (Sim, 2018). The focus of this article is on the use of checkpoint inhibitors to treat some types of advanced urological and lung cancers.

Militaristic metaphors and the language of war permeate biomedical discourse on the immune system, emphasizing fixed dichotomies across relations such as self/other and internal/external (e.g., Cohen, 2009; Haraway, 1991; Huang, 2014; E. Martin, 1990, 1994; Napier, 2002). The notion of the body as ‘defended nation-state’ that requires protection from external forces dominates the discourse in immunology, grounding a representation of the body as an ‘embattled self’ (Jamieson, 2015, p. 108). This notion of ‘immunity-as-defence’ gained traction starting in the mid-nineteenth century, and has been discussed by scholars examining the intersections between conceptualizations of the ‘self’, the body, and identity in areas such as cancer and HIV/AIDS (Haraway, 1991), antimicrobial resistance (e.g., Cohen, 2009; Davis et al., 2016; E. Martin, 1994; Napier, 2002), transplantation, and blood donation (Brown, 2019; E. Martin, 1994). The use of warfare or policing metaphors that frame T-cells as soldiers, or on patrol protecting internal borders, are also well established, each contingent upon and entwined with contemporary political moments (Cohen, 2009; E. Martin, 1990, 1994). Entangled with these militaristic metaphors are framings of the immune system as agile, adaptable, and resilient (Napier, 2002).

In Euro-American contexts, militaristic metaphors pervade discourses around cancer survivorship, too, in which patients battle against the disease and a hope of overcoming, beating, or surviving the battle (Bell, 2013; Jain, 2013; Sontag, 1979; Stacey, 1997). Such metaphors offer the possibility of heroic struggles in the face of disease, avoiding viewing cancer patients as victims (Bell, 2012). Such dominant cultural images of cancer survivorship often overshadow accounts of sadness, chaos, and disruption (Frank, 2013).

The social science literature on the discourses of immunology and cancer focuses on semiotic practices while also attending to the social and political stakes of such metaphorical framing (Kazimierczak & Skea, 2015; A. Martin, 2010; Reisfield & Wilson, 2004; Sontag, 1979; Stacey, 1997). Scholars have examined the use of military metaphors in representing both cancer survivorship (Ross et al., 2019) and immune function, and in cementing fixed boundaries around self/non-self (e.g., Haraway, 1991; Jain, 2013; A. Martin, 2010; Sontag, 1979; Stacey, 1997). Sontag (1979) argues that metaphors draw the illness away from its concrete, ordinary, and political manifestations, obfuscating the experience of disease. Building on Sontag’s work, Stacey (1997), and more recently Jain (2013), have shown how metaphors go beyond ‘literary tropes’ and shape notions of gender, health, and identity, with Jain and Stacey (2015, p. 5) arguing that ‘cancer is not a noun, but a conglomeration of interests’. Both Jain (2013) and Stacey (1997) investigate such metaphors and their social and political stakes, claiming that it is through their construction that cancer is rendered meaningful. Such metaphorical framing in cancer, which cements fixed boundaries around self/non-self, has also been connected to wider expectations for individuals to take responsibility for and optimize their health and sense of self, through practices of self-governance, including so-called ‘lifestyle’ choices such as diet and fitness (Bell, 2009; Rose, 2007).

For Jamieson (2015), militaristic imagery ‘promotes a view of the organism as a defended, discrete, biological entity that is only capable of interacting with others in violent or antagonistic ways’ (p. 108). Such violent and antagonistic relations between self and non-self (either in terms of one’s own body or the wider body politic) contributes to ‘a politically invested concept of self—namely, an isolated, autonomous individual that exists within, but separately from, an environment of others’ (Jamieson, 2015, p. 108) authorizing a political subjectivity which, for example, asserts war as a ‘natural’ order of life (E. Martin, 1990, p. 417). More recently, however, feminist STS scholars have troubled self/non-self distinctions, suggesting that images of primarily antagonistic relations do not align with ‘biological thinking about how organisms coexist in shared ecologies, sometimes with great mutual benefit, sometimes pacifically, sometimes indifferently, and sometimes deleteriously’ (Cohen, 2009, p. 8). For example, organ donation (Shildrick et al., 2010), the ‘cell-trafficking’ between fetal and maternal bodies (A. Martin, 2010), and the bacteria that cohabit our gut (Lorimer, 2016; Waldby & Mitchell, 2006) demand alternatives to antagonism.

## Troubling self/non-self: Discourse and materiality

In recent years, feminist STS scholarship in immunology has pointed to a shift away from militaristic metaphors toward more complex framings of disease that disrupt the longstanding self/non-self dichotomy, and that pay attention to materiality. Scholars attend to the interwoven effects of the material and semiotic, troubling the fixed boundaries around self/non-self and emphasizing how the body has the potential to exceed scientists’ characterizations (A. Martin, 2010), ‘surprise’ us (Shildrick, 2010), or ‘kick back’ (Barad, 1998). As A. Martin (2010, p. 40) suggests, ‘experimental surprises are clues to nature’s agency’, where the binary distinction between self and non-self does not necessarily ‘account for the surprises that the body has in store for us’ (Shildrick, 2010, p. 18). In this way, ‘nature asserts itself in spite of dominant tropes’, refusing to ‘act in the ways predicted by existing frames’ (A. Martin, 2010, pp. 25–26). A. Martin’s (2010) work on fetal-maternal microchimerism represents a shift away from antagonism and identity and difference (p. 46; see also Colls & Fannin, 2013; Matzinger, 2002). Martin argues that the cell-trafficking between the foetus and pregnant body serves as a challenge to the antagonism that underpins immunology discourse and calls instead for the need to pay attention to ‘productive relations of self and other’ through attending to functionality and relationality. For Davis et al. (2016, p. 135) in their work on antimicrobial resistance, ‘this erosion of the absolute in [self/non-self] implies … “immune-cosmopolitanism,” that is, an understanding of one’s immunity as reliant on productive relations and amicable coexistence with the other’ (see also Shildrick, 2014).

In emphasizing ‘nature’s agency’, feminist STS scholars attend to the liveliness of biological being, moving beyond discourse to consider what the immune system does rather than what it represents (Colls & Fannin, 2013; Fannin, 2014). In so doing, they are responding to what new materialists would consider the exclusion of materiality when language, discourse and culture are overemphasized (Barad, 2007; Fannin, 2014).

In the context of cancer, Kazimierczak and Skea (2015) shift analytical attention away from metaphor as ‘purely rhetorical form’ and instead explore the discursivity of cancer through specific material and semiotic relations and practices. Discourses are not solely consigned to the sphere of language but are ‘particular arrangements of conceptual and material relations and practices, where concepts are always already material as well as semiotic, and materialities bear various meanings’ (Kazimierczak & Skea, 2015, p. 341). The material and the semiotic are mutually constitutive and inextricably linked, constituted through relations, or ‘intra-action’ (Barad, 2003). Troubling the longstanding self/non-self dichotomy, and emphasizing the ‘liveliness’ of the material world is part of a broader commitment to bringing into being new social and political realities by demonstrating that the ‘biological’ is not fixed (A. Martin, 2010). As A. Martin (2010, p. 46) and others suggest, ‘the biological body props up the body politic’ where what is deemed ‘natural’ is used to ‘justify visions of social organization’ by cementing binary distinctions, for example, male/female.

In the context of cancer and immunology, discourse around immunotherapy, both cements the antagonism associated with self non-self relations where it is framed as an internal battle, and also troubles these antagonistic relations through emphasizing the body’s capacity to heal and framing it as less harmful on the body. This shift in discursive framing opens up possibilities for patients’ embodied experiences of cancer, generating new forms of hope. At the same time, this shift has the potential to generate new forms of embodied labour since it positions the individual patient’s body as key to the healing process: Paradoxically (re)enrolling the body in the ‘fight’ against cancer.

In this article I argue that it is critical to attend both to framings on a metaphorical level and to material stakes of how framings shift. We should pay attention to embodied experiences (Barad, 2007; Blackman, 2012; Haraway, 1988) and the body we do (Mol & Law, 2004, p. 51) in generating ‘knowledge-in-practice’ in the context of immunology. This commitment to theorizing embodied and lived experience is part of a wider commitment to moving STS analysis of immunology outside the laboratory with an emphasis on what metaphorical framing and discursivity in cancer and immunology is doing as well as representing. Overall, this article contributes to feminist STS literature that troubles the antagonism associated with self/non-self relations, demonstrating that metaphors are not ‘wholly determinative’ (A. Martin, 2010, p. 40), and STS literature which theorizes the entanglements between discourse and materiality including emergent tensions (see Barad, 2003; Giraud, 2019; Hollin et al., 2017; Murphy, 2012).

## Methods

This article draws on semi-structured qualitative interviews carried out with practitioners and patients across a cancer service in NHS Scotland. The majority of the data drawn on in this article are taken from semi-structured interviews.

I carried out ten interviews with practitioners, including clinical nurse specialists, research nurses, and medical and clinical oncologists. I also interviewed ten patients across the lung and urology cancer service teams (three patients in the urology cancer service and seven patients in the lung cancer service). Immunotherapy is prescribed as standard of care treatment for non-small cell lung cancer (a specific classification of lung cancer) and urological cancers, including renal cancer. A number of immunotherapy-based clinical trials in the adjuvant setting (post-surgery) were also conducted across the research site and with the cancer teams. Eight patients were living with advanced cancer and were being treated with standard of care immunotherapy, and two patients were enrolled on immunotherapy clinical trials to assess the efficacy of immunotherapy for preventing cancer recurrence. Patients and practitioners were recruited with the aid of a specialist nurse gatekeeper. The specialist nurse introduced me to each clinical team and from there I approached practitioners directly. The specialist nurse also approached suitable patients undergoing treatment in out-patient clinics on my behalf and I then contacted individuals directly to provide further information about the project and to seek consent where appropriate. Pseudonyms are used throughout the article.

Interviews were semi-structured and were carried out between March 2021 and October 2021 (fieldwork is on-going) and lasted between 60 and 90 minutes. During interviews with practitioners, questions centred on how they described immunotherapy to patients in clinical practice (direct observations of those scenes were not possible, due to restrictions imposed by the ongoing Covid-19 pandemic) with an emphasis on the challenges and opportunities associated with novel immunotherapy treatments for clinical practice. These included managing the expectations and hopes that patients attached to these novel therapies as well as managing uncertainties around side effects and toxicities, with a specific emphasis on how checkpoint inhibitors differed from other therapies, such as chemotherapy and radiotherapy. During interviews with patients, questions centred on how practitioners approached patients about these therapies, their views and perspectives on the role of the immune system in cancer and how they dealt with their treatment, including managing side effects, toxicities and uncertainty. I adopted a situational analysis approach to analyse interview transcripts thematically and discursively, and I dealt with data manually (see Clarke et al., 2016). Across the transcripts I traced the use of metaphor (e.g., use of militaristic metaphors) and analysed how this subtly shifted. In line with the conceptual focus of the article, and attention to both discourse and materiality, I also traced the day-to-day practicalities of treatment as I asked patients to reflect on how these therapies affected their daily lives. A combination of deductive and inductive approaches were therefore used as I sought to interrogate the data based on readings of the literature alongside adopting a situational analysis approach. In line with feminist STS scholarship, I paid attention to the tensions provoked by the wider promissory and discursive framing of these novel therapies (Haraway, 1988; Jain, 2013; Murphy, 2012; Puig de la Bellacasa, 2011).

I begin the analysis by focussing on practitioners’ framing of immunotherapy, which is consistent with the discourse concerning immunology and cancer more widely. The immune system does not recognise cancer and therefore evades the self versus non-self distinction that pervades immunology discourse more widely. The purpose of immunotherapy treatment is therefore to restore the ‘natural’ functioning of the immune system to recognise cancer as non-self, which practitioners emphasized during interviews. To do so, they adopted the use of militaristic metaphors and metaphors, which draw on fantasy and speculative fiction, to cement boundaries around self/non-self and depict an internal battle. However, the discursive framing of immunotherapy also subtly shifts: While immunotherapy restores the ‘natural’ functioning of the immune system to recognize cancer, it is also framed as ‘gentle’ on the body and the body’s own capacity to heal is emphasized. Emerging from this is a more dynamic and less antagonistic version of the relations between self/non-self (the immune system and cancer).

I then go on to explore the material consequences of this framing by highlighting patients’ embodied labour, with an emphasis on the ‘body we do’ (Mol & Law, 2004) when patients are actively enrolled in treatment. The subtle shift in discursive framing that places emphasis on the body as an active agent in disease treatment creates new forms of labour for patients and materializes particular tensions around the day-to-day practicalities of cancer treatment. Treatment practices to sustain and maintain life are demanding, as patients are faced with dealing with clinical uncertainties concerning response rate, toxicity, and prognosis. New and individualized ontologies of the self are built around the need to establish a ‘healthy’ immune system to manage advanced cancer over time. I argue that immunotherapy refigures the antagonistic relations between self and non-self, as cancer treatment is described as ‘kinder’, ‘gentler’ and ‘more tolerable’. Yet this shift in discursive framing also generates tensions in the form of new and embodied forms of patienthood that further individualize and enrol patients in the ‘fight’ against disease.

## Findings

In an interview, a medical oncologist involved in immunology and cancer research explained how the relationship between cancer and the immune system escapes the self/non-self model that underpins the immunology and cancer discourse more widely,

In theory, cancer shouldn’t be possible if our immune system was fully functional, because all cancer cells are non-self; they are all by definition different from self cells …

Cancer must somehow escape from that [recognizing self from non-self] and there’s lots of different potential ways to do it. So I guess the best example of how the immune system is taught not to attack non-self is when someone’s pregnant and able to switch that off. There’s a general process called anergy where your body decides whether it needs to be worried about something—that could be sequences on cells or proteins, for example proteins you encounter when you eat your food, bacteria in gut … there are lots of different potential ways for the cancer to hide from a system that really should be able to attack it and clear it. (Medical oncologist)

The relationship between cancer and the immune system evades the fixed binary distinction of self versus non-self: cancer emerges and spreads because the immune system fails to detect it as non-self. These practitioners point to other ways in which the immune system ‘naturally’ elides the fixed self/non-self distinction, for example, in pregnancy. Immunotherapy is framed by practitioners as key to restoring the immune system to its ‘natural’ state (see Colls & Fannin, 2013; Fannin, 2014): re-establishing the self/non-self dichotomy by reengineering the immune system to detect non-self (cancer).

Analysing practitioners’ accounts where they detailed how they explained these novel immunotherapy treatments to patients in the clinic, they placed emphasis on restoring the immune system to recognize ‘non-self’: ‘boosting’ the immune system, or as a consultant oncologist explained, ‘waking up’ the immune system,

I suppose I sometimes talk about the fact that generally cancers are quite good at hiding from the body’s immune system, and so that’s leading into this kind of use of the term, ‘waking it up’.

Practitioners also constructed boundaries around self and non-self (cancer) in their explanations to patients. To do so they drew primarily on militaristic metaphors. Positioning the immune system as key to the ‘fight’ against cancer was a well-rehearsed trope across practitioners’ accounts, as highlighted in an interview with a consultant oncologist:

Releasing your immune system from the suppression caused by the cancer, which then reactivates your immune system to fight against the cancer.

The battle against cancer persists via the use of militaristic metaphors and through the discursive framing of T-cells as soldiers and the immune system as the army, as another consultant oncologist explained:

It’s talking about those things, about boosting the immune system, about boosting a specific part of your immune system. We talk about the army and we want to boost these soldiers rather than those soldiers and at the moment those poor soldiers can’t see the enemy because they’ve got an invisibility cloak and we need to take the invisibility cloak away.

That’s what we ideally want, once we’ve taken that away, the soldiers can see.

Warfare metaphors were used in the clinic to cement and draw boundaries around self/non-self. At the same time, they were used inconsistently, and other metaphors which drew on fantasy imagery or speculative fiction were adopted. Just above, the consultant oncologist referred to an ‘invisibility cloak’, which prevents the immune system from targeting cancer. This particular use of metaphor also emerged during an interview with another consultant oncologist, who remarked, ‘I sometimes will use the analogy of the invisibility cloak for Harry Potter and taking off the invisibility cloak and exposing the cells to the immune system.’ Discourse around immunotherapy maintains the self/non-self binary distinction: re-engineering the immune system to ‘fight’ cancer or to enable the immune system to see ‘the enemy’. Both military metaphors and others often reinforce the self/non-self distinction in connection with the immune system.

This shifting use of metaphor, or situated and contextual use of language, opens up possibilities for other ways of framing immunotherapy that trouble the antagonism associated with self/non-self distinctions. In the following section, I analyse how practitioners and patients frame immunotherapy through an emphasis on the body’s capacity to heal. This offers a ‘provocative alternative’ (A. Martin 2010, p. 42) for patients in the form of hoped for treatment success.

### Immunotherapy and the ‘capacity to heal’

Despite practitioners using metaphors that reinforce the self/non-self distinction, and that reinstate sharp boundaries, they also framed immunotherapy in terms of the body’s capacity to heal itself, and as being less harmful on the body than are alternatives. This discursive shift troubles the antagonism associated with self/other relations that pervade wider immunology discourse, and the use of metaphor, as it emphasises immunotherapy as ‘gentle’ on the body, rather than an internal battle. During an interview, a clinical nurse specialist working in the lung cancer service explained how she described the treatment to patients:

I suppose what I often say to patients is it kind of uncloaks the cancer, so it kind of stops the cancer from hiding from the immune system and enables the body to then actually help to get rid of the cancer, I suppose that’s very layman terms of …

Researcher: You used this phrase ‘uncloaks cancer’. Why do you use this particular phrase?

Yes, because when you try and explain immunotherapy people say, ‘well why doesn’t the body do that already?’ You know, ‘why doesn’t the immune system find the cancer if that’s what it is meant to do? It’s meant to kind of clear up, you know, dead cells or unwanted cells or infections, why doesn’t it do that already?’ You know, I will say to people the immune therapy drug … activates the immune system, so it uncloaks the cancer, because it just then gives the impression of the cancer hiding from the immune system, so we kind of use that term … so it tends to be, you know, a little bit gentler or more tolerable. (Clinical nurse specialist)

This nurse drew on language which continues to draw on the self/non-self model (conjuring images of uncloaking cancer, as above) in order to explain to patients how cancer escapes the immune system. As she iterated, this can be difficult for patients to comprehend, given that the immune system and its role in managing disease is culturally pervasive (see Davis et al., 2016). However, as evidenced in her account, this particular explanation of immunotherapy marks a shift away from antagonism (internal battle). Immunotherapy reinstates the self/non-self model, but at the same time, also refigures antagonism as it is described as ‘gentler’ or more ‘tolerable’ than other cancer therapies. Describing immunotherapy in this way provides a ‘provocative alternative’ (A. Martin, 2010, p. 42) to the ‘suffering’ attached to discourse around other forms of cancer therapy such as chemotherapy (Bell, 2009).

This discursive shift towards more dynamic and less antagonistic immune system/cancer relations (Davis et al., 2016, p. 135), ‘you know, it’s trying to have a language that things inside you that are actually helpful’ has the potential to bring into being ‘provocative alternatives’ (A. Martin, 2010, p. 42) in the form of new ways of experiencing cancer treatment. A research nurse reflects,

I think what drives people’s hope is the fact that they’re helping themselves, you know their own immune system is possibly doing the work … psychologically your own immune system working to help you, I think gives them [patients] a bit of a boost.

When patients come to clinic and we talk about toxicities, and we do a thyroid check and perhaps things aren’t working as they should be, patients mention that actually, this shows that the immune system is working and they’re quite pleased about this.

Subtle shifts in discursive framing open up possibilities for new forms of patienthood. Patients’ own bodies are positioned as key to the healing process, creating new configurations of hope and ambivalence attached to treatment. For the nurse quoted just above, this includes the ambivalence around treatment side effects, where (unwanted) side effects might provide hope for patients as they ‘show that the immune system is working.’

During interviews, patients reflected on their understandings and perceptions of immunotherapy as a new and novel cancer treatment. The ‘self fighting non-self’ dialectic pervades their framing of the treatment, reflecting widespread cultural notions of strengthening the immune system to help the body ‘fight’ disease (Davis et al., 2016). The distinction is subtly refigured to account for the body’s capacity to heal. Patients David and Rachel explained that this provides hope:

Well it just made sense that if you can stimulate the body’s own immune system to fight off a … the foreign cells as it were, it just makes … you know, logically if you think about it, if you can stimulate the body’s own immune system to fight against a foreign body as it were, then that makes sense to me at least. If the immune system is so effective in other aspects of … you know, you cut your finger and your immune system heals you, you know, nine times of ten. The body itself’s healing. (David)

Immunotherapy opens the key and lets the body fight cancer cells. (Rachel)

Both patients drew on the widespread cultural notion that the immune system ‘fights’ disease in order to make sense of this new cancer therapy. The self/non-self distinction is maintained, but the antagonism associated with it is refigured. As patients Olivia and Richard discussed in their accounts of immunotherapy,

That’s how I would look at it. It’s helping your body fight whatever it has to fight and immunotherapy is just basically telling you what it is, isn’t it? It’s helping your immune system fight your cancer maybe so that’s how I look at immunotherapy. It’s giving you the extra chance just to … or giving you something else, a wee bit more support to fight it, sort of thing. I think that’s what your body to sort of try and cure itself sort of thing or help itself sort of thing. (Richard)

They said that it was to boost my immune system to deal with the cancer so that my body would be doing the work, but the immunotherapy was to boost my system to do that. (Olivia)

For these patients, immunotherapy is mobilized as a key actor in the ‘fight’ against cancer. However, patients’ own bodies are also positioned as active agents in the healing process, with the language of cure also circulating in Richard’s account.

Continuing the interview with Olivia, I asked her to elaborate on what she meant by ‘my body doing the work’ and she became very emotional.

I tried to think about it as … not as an alien to be got rid of, but just as part of me that had gone a bit wrong. I thought of it quite kindly in a way, as part of me, it needs to be sorted out, but it’s not, ‘oh, go away, get out of me’, sort of thing … it’s not an alien, it’s me. I do feel that immunotherapy is much more holistic. It’s certainly not poisonous in the same way. It’s kinder to the functioning of your body. It doesn’t feel as if you’re under attack, and I think … I don’t think I just mean it in terms of it’s a physical thing, I think I mean it more emotionally … it’s, sort of, you know, it just … it just feels like a kinder thing to do really. (Olivia)

Here we see the antagonism that underpins immunology discourse refigured and resisted as patients’ discourse around immunotherapy shifts to deemphasize treatment as an internal battle. ‘It doesn’t feel as if you’re under attack’, which for some patients provides hope. Olivia’s framing of immunotherapy as ‘kinder’ both physically and emotionally, which echoes the clinical nurse specialist I interviewed, involved contrasting this novel therapy with the suffering attached to other cancer therapies such as chemotherapy or radiotherapy (Bell, 2009). As Rachel also noted, *‘*it feels great that you can actually do something and that will help my body fight it, which it had apparently given up on’.

In these accounts the discursive emphasis on the capacity to heal works to materialize particular configurations of cancer treatment as ‘kinder’, with the body positioned as an active agent in this process. We also witness shifting cancer ontologies: Cancer is not necessarily othered as a monstrous entity or ‘alien’ figure residing in the body but is difficult to separate from self (Jain, 2013). These discursive shifts trouble the antagonism associated with self/non-self relations (treatment encompassing an internal battle) and emphasizes function and productive co-existence (Davis et al., 2016, p. 135). In the final section, I develop the analysis to explore how shifts in discursive framing and language trouble the antagonism associated with self/non-self relations, but also materialize particular tensions for patients living with and managing advanced cancer. In so doing I emphasize the tensions at work in specific configurations of the entanglements between discourse and materiality (see Giraud, 2019; Hollin et al., 2017; Murphy, 2012).

### Material stakes of discursive shifts: Emergent tensions

In patients’ accounts of treatment, emphasis on the body’s capacity to heal materialized tensions for patients living with and managing advanced cancer. These included managing on-going side effects and toxicities and engaging in activities that further individualize bodies as part of establishing a ‘healthy’ immune system—which required a significant amount of embodied labour in the ‘body we do’ (Mol & Law, 2004). Exactly what counts as self was also called into question as patients become enrolled in on-going care of the self over time (see Davis et al., 2016, 2023). The tensions around the experiences of treatment side effects and toxicities, were particularly present in Helen’s account of immunotherapy:

He said about the therapy attacking the good cells in the body and sometimes that can damage things in the body, so you know, there is a good and a bad side of it. … yes, it takes a wee while to get your head round it as well, you know, that you know, this stuff is going in your body, you know, and what it does, it’s quite difficult to understand, and it’s now, it has attacked my thyroid gland which has just made me feel ill.

I don’t know the word to use. It’s got some sort of lighter effect apart from the thyroid. Well, one nurse, she is a lovely girl, and she sat down one day when she was putting my intravenous in, and she used to say, now think of it as we’re putting little piranha fish into your body and they are going to go around and eat all the cancer and the bad cells and things like that. It was quite funny, but I thought it was quite good as well … And … sometimes they’ll nibble at wrong things, which like my thyroid, you know. (Helen)

The framing of immunotherapy that Helen described as ‘supposed to make you feel better’ creates tensions in terms of her experiences of treatment and the practicalities of doing immunotherapy day-to-day (Mol, 2008; Mol & Law, 2004). Immunotherapy was also framed by Helen’s practitioner as ‘having a good side and a bad side’. This imagery sits uncomfortably alongside the numerous ways in which immunotherapy is also framed as having a ‘lighter effect’ or is ‘kinder’, ‘gentler’ or ‘more tolerable’. The discursive framing of immunotherapy as ‘kinder’ or ‘more tolerable’ and here ‘lighter’ is in tension with the experiences of patients in terms of the day-to-day practicalities of treatment: Marks are left on bodies (Barad, 2003, p. 828).

During an interview with Rachel, who was diagnosed with advanced renal cancer, she gave an insight into the tensions at work and the day-to-day practicalities of undergoing immunotherapy treatment.

And in terms of side effects, I’ve had most of them … The worst one obviously is the fact it knocked my pancreas out so from my point of view that’s the worst, although probably the easiest for them to manage. But no, every few months you would go and have treatment and something else would be wrong with your bloods, but we kept going and it kept working. It could be quite depressing in as much as you just wondered what was going to go wrong. My GP said I had sepsis at one point that I was admitted for, but I think it was pretty minor, I just had some IV antibiotics and stayed in for a week. My cortisol levels were wrong, my thyroid went wrong at one point.

And I think that’s exactly what I’ve done, I’ve been really lucky because when my body’s reacted it’s come back down again within two weeks. I’ve tolerated it and it’s got back to normal and we’ve worked through it. Yes, I mean, every time you go you learn something new about it. And it is amazing and I don’t completely understand how it works but I wouldn’t be here, I don’t think without it. (Rachel)

Frontstage, Rachel is reconciled with the idea that what ails the body can also be also used to treat the body. However, the demands placed on her to persist with treatment position the body as active agent in the healing process, even though it can be extremely toxic and harmful. The discursive framing of the body’s capacity to heal has the potential to mask or elide the demands of these treatments on patients’ bodies, as well as patients’ embodied labour in a context of hoped-for treatment success for advanced cancer. Rachel spoke at length about her experiences of side effects and of the physical demands of treatment. She also described herself as ‘lucky’ for being able to ‘tolerate’ the treatment, highlighting the discursive shift towards healing, but the material consequences of which involves new forms of embodied labour over time. She emphasized the need to ‘keep going’ in the face of the debilitating side effects brought on by her body’s reaction to treatment. This sense of resilience evoked in Rachel’s account reflects an individualizing logic undergirding the discursive portrayal of the body as active agent in disease treatment, requiring patients to engage in ongoing care of themselves over time (see Cohen, 2009). All of the patients I interviewed described how they engaged in a wide range of practices to establish a ‘healthy’ immune system, such as maintaining hydration (particularly among those diagnosed with renal cancer), minimizing alcohol intake, maintaining a ‘healthy’ diet, and exercising where possible.^3^

Adrian, who was diagnosed with advanced kidney cancer, discussed his expectations concerning immunotherapy, and reflected on the fact that while it may not necessarily lead to a ‘cure’, there were additional things he could do to maximize the chance of treatment success:

Like I say, it’s not going to cure it, but it’s going to help, keeping yourself fit, you know. Eating the right stuff, fruit and veg and that. I drink a lot of water, you know. I think it’s important that you, if you’re able to, have a walk. I do it early morning and that’s it done. You feel better after it, you know.

Similarly, Mark outlined the importance of remaining active and independent when discussing how the immunotherapy had begun to shrink the tumours on his lungs.

So the immunotherapy must be doing something, like, you know. Or something’s happening somewhere. … it was basically like using your own immune system to help your body basically. [H]e said [consultant] that he’s surprised that I am actually still here and he was going on about, have you gone out walking and keeping fit, and I was like, yeah. … But when I think about it, I'm like, there must be a lot of people out there that do actually live in a family environment or live with a wife and when they get this diagnosis, they’ve got someone there that’s always, ‘Oh I’ll do this for you, I’ll do that for you’. They’re not being so independent and are just sitting there. I don’t know, it’s just a theory of my own, like, that maybe that I’m having to do or I'm making myself do a lot more for myself that’s helping this immunotherapy fight it inside my body.

While the immune system is given agency, for treatment to be successful, ongoing bodily labour is necessary, here in the form of remaining active and independent.

As evidenced in these interview excerpts, the individualizing logic at the centre of wider immunology and cancer discourse is entrenched in patients’ accounts of treatment. By deemphasizing antagonism (internal battle), the discursive emphasis on the body’s capacity to heal troubles the importance of the self/non-self distinction. But paradoxically individualized bodies are still enrolled in the ‘fight’ against cancer. As Mark further explains,

‘Cause your immune system will always fight as much as you’re fighting basically, I think. … I think you would have to put some effort in as well, like, you know … It is a real challenge, like. This week was one of the worst.

But … rather than just sit about the whole day, just … it’s not good for you. Not good for you in a normal circumstance, never mind circumstance where your body’s trying to fight something.

Mark perceives bodily work to be demanding but necessary for treatment success. For Richard, this involved working alongside practitioners: ‘My part to play is just keeping myself fit and healthy and let the doctors do their part’. Patients often described feeling obligated in this, seeing it as encompassing a ‘full-time job’.

Some people say, well, you wouldn’t … you know, the immunotherapy has worked well, because I’m into sport, and stuff, but, you know, I think it all complements each other. … It’s just trying to find a balance of everything, you know, you can imagine … if you’ve got a plate and you put your finger in the middle of the plate and on the edge you’ve got weights and the weights are things, like, nutrition or sleep, work, exercise, you know, you do too much of one thing, the whole plate upsets or if you do too less, you know. It’s just trying to keep that balance the whole time and it’s quite a full-time job to keep it all, you know, just right, just keep it all spinning around and around, you know. (Jack)

Jack was diagnosed with advanced lung cancer and was enrolled in an immunotherapy clinical trial. He described a number of side effects that he experienced, including colitis and pancreatitis, for which he had to be hospitalized. He described the importance of achieving balance in terms of a healthy lifestyle to maximize the chance of treatment success but at the same time reflected on how difficult this was to carry out in practice. He directly credited this lifestyle with establishing a ‘healthy’ immune system. ‘I’ve always had a really good immune system, I never get ill, you know, I never get colds or anything and I thought, well, that’s [immunotherapy] got to be worth a chance’.

In this section, I have begun to explore the tensions that arise when discursive framing around immunotherapy positions the body as active agent in treatment, generating new forms of embodied labour. This includes the ongoing management of treatment side effects or toxicities. This discursive shift troubles the antagonism associated with the self/non-self distinction by emphasizing kinder, gentler therapies, but results in further individualizing bodies in the ‘fight’ against cancer as patients are engaged in eating healthily, taking vitamins, and exercising to ‘boost’ their immune system and support its natural function (Mol & Law, 2004).

## Discussion

In this article I have developed a feminist STS analysis that troubles the longstanding antagonism associated with the self/non-self distinction. Through examination of patients’ experiences, I have highlighted shifts in discursive framings and the material stakes of these shifts where discourse around immunotherapy has the potential to bring into being new forms of embodied patienthood,

In the first section of the analysis, I focused on how practitioners framed immunotherapy as key to restoring the ‘natural’ functioning of the immune system, and the self/non-self model: Cancer is possible because the immune system fails to recognize cancer cells, but immunotherapy allows that recognition. However, the self/non-self model underpinning immunology and cancer discourse fails to account for the ‘surprises our bodies have in store for us’ (Shildrick, 2010, p. 18). Practitioners’ accounts drew on militaristic metaphors and imagery tied to fantasy and speculative fiction to frame immunotherapy and reemphasize the self/non-self dichotomy. The shifting use of metaphor opened up other discursive possibilities in the context of immunotherapy. This included emphasizing the body’s capacity to heal where the body is positioned as active agent in disease treatment and as ‘gentle’ and ‘tolerable’: challenging the antagonism associated with self/non-self relations (see Davis et al., 2016). I then developed the analysis to examine how the discursive framing of immunotherapy as ‘kinder’, ‘gentler’ ‘more tolerable’ (deemphasizing antagonistic self/non-self relations) brought into being new material realities for patients in the form of hoped for treatment success. Further analysing patients’ accounts however, this subtle shift in discursive framing to emphasize treatment as less harmful on the body also materialized tensions, generating new and demanding forms of labour in the day-to-day management of cancer. Discursive shifts reveal not only that metaphors are not ‘wholly determinative’ (A. Martin, 2010, p. 40) but that tensions emerged in specific configurations of the entanglements between discourse and materiality: emphasizing the capacity to heal further individualized patients’ experiences of cancer (see Giraud, 2019; Hollin et al., 2017; Murphy, 2012). Discourse and metaphors are therefore not neutral—‘marks are left on bodies’ (Barad, 2003, p. 828) and tensions stem from the entanglements between meaning and matter (see Giraud, 2019).

While feminist STS literature emphasizes the need to pay attention to the liveliness of biological being and productive coexistence, offering ‘provocative alternatives’ for being in the world this framing also presents tensions in the context of cancer. Emphasis on the capacity to heal has the potential to enact or materialize tensions, as patients take on care of themselves over time in order to live (well) for longer with cancer. They are left with the bodies they ‘do’ (Mol & Law 2004). Not only then is the self/non-self model refigured, but new and individualized ontologies of the self emerge as patients’ bodies become enrolled in therapy to maximize treatment success and to realise the hopes attached to novel immunotherapy treatments. In so doing, this framing further individualised bodies and treatment experiences, eliding the practical and emotional demands placed on patients (Frank, 2013), many of whom have foreshortened futures.

## References

[bibr1-03063127231199217] Barad K. (1998). Getting real: Technoscientific practices and the materialization of reality. Differences: A Journal of Feminist Cultural Studies, 10(2), 87–128.

[bibr2-03063127231199217] Barad K. (2003). Posthumanist performativity: Toward an understanding of how matter comes to matter. Signs, 28(3), 801–831.

[bibr3-03063127231199217] Barad K. (2007). Meeting the Universe Halfway – Quantum physics and the entanglement of matter and meaning. Duke University Press.

[bibr4-03063127231199217] Bell K. (2009). ‘If it almost kills you that means it’s working’: Cultural models of chemotherapy expressed in a cancer support group. Social Science & Medicine, 68, 169–176. 19010578 10.1016/j.socscimed.2008.10.023

[bibr5-03063127231199217] Bell K. (2012). Remaking the self: Trauma, teachable moments and the biopolitics of cancer survivorship. Culture, Medicine & Psychiatry, 36(4), 584–600. 23054293 10.1007/s11013-012-9276-9 PMC3496522

[bibr6-03063127231199217] Bell K. (2013). Biomarkers, the molecular gaze and the transformation of cancer survivorship, Biosocieties, 8(2), 124–143. 23750174 10.1057/biosoc.2013.6 PMC3671370

[bibr7-03063127231199217] Blackman L. (2012). Immaterial bodies: Affect, embodiment, mediation. Sage.

[bibr8-03063127231199217] Brown N. (2019). Immunitary life: A biopolitics of immunity. Palgrave Macmillan.

[bibr9-03063127231199217] Canavan N. (2017). A cure within: Scientists unleashing the immune system to kill cancer. Cold Spring Harbor Laboratory Press.

[bibr10-03063127231199217] Cancer Research UK. (2021, May). Checkpoint Inhibitors. Retrieved October 12, 2021, from https://www.cancerresearchuk.org/about-cancer/cancer-in-general/treatment/immunotherapy/types/checkpoint-inhibitors

[bibr11-03063127231199217] Clarke A. E. Friese C. Washburn R. (2016). Situational analysis in practice: Mapping research with grounded theory. Routledge.

[bibr12-03063127231199217] Cohen E. (2009). A body worth defending: Immunity, biopolitics, and the apotheosis of the modern body. Duke University Press.

[bibr13-03063127231199217] Colls R. Fannin M. (2013). Placental Surfaces and the Geographies of Bodily Interiors. Environment and Planning A: Economy and Space, 45(5), 1087–1104.

[bibr14-03063127231199217] Davis M. Flowers P. Lohm D. Waller E. Stephenson N. (2016). Immunity, biopolitics and pandemics: Public and individual responses to the threat to life. Body & Society, 22(4), 130–154.

[bibr15-03063127231199217] Davis M. Lohm D. Flowers P. Whittaker A. (2023). The immune self, hygiene and performative virtue in general public narratives on antibiotics and antimicrobial resistance. Health, 27(4), 491–507. 34541910 10.1177/13634593211046832

[bibr16-03063127231199217] Fannin M. (2014). Placental relations. Feminist Theory, 15(3), 289–306.

[bibr17-03063127231199217] Finck A. Gill S. I. June C. H. (2020). Cancer immunotherapy comes of age and looks for maturity. Nature, 11, 3325. 10.1038/s41467-020-17140-5 PMC7335079 32620755

[bibr18-03063127231199217] Frank A. (2013). The wounded storyteller: Body, illness, and ethics. University of Chicago Press.

[bibr19-03063127231199217] Giraud E. H. (2019). What comes after entanglement?: Activism, anthropocentrism, and an ethics of exclusion. Duke University Press.

[bibr20-03063127231199217] Haraway D. (1988). Situated knowledges: The science question in feminism and the privilege of partial perspective. Feminist Studies, 14(3), 575–599.

[bibr21-03063127231199217] Haraway D. (1991). Simians, cyborgs and women: The reinvention of nature. Routledge.

[bibr22-03063127231199217] Huang S. (2014). The war on cancer: Lessons from the war on terror. Frontiers in Oncology, 4(293), 1–4. 25368844 10.3389/fonc.2014.00293 PMC4202687

[bibr23-03063127231199217] Harris L. A. (2018). Collection on cancer immunotherapy: Editorial. British Journal of Cancer, 112.

[bibr24-03063127231199217] Hollin G. Forsyth I. Giraud E. Potts T. (2017). (Dis)entangling Barad: Materialisms and ethics. Social Studies of Science, 47(6), 918–941. 28914174 10.1177/0306312717728344

[bibr25-03063127231199217] Jain S. L. (2013). Malignant: How cancer becomes us. University of California Press.

[bibr26-03063127231199217] Jain S. L. Stacey J. (2015). On writing about illness: A dialogue with S. Lochlann Jain and Jackie Stacey on cancer, STS, and cultural studies. Catalyst: Feminism, Theory, Technoscience, 1(1), 1–29.

[bibr27-03063127231199217] Jamieson M. (2015). The politics of immunity: Reading Cohen through Canguilhem and new materialism. Body and Society, 22(4), 1–24.

[bibr28-03063127231199217] Kazimierczak K. A. Skea Z. (2015). ‘I’ve used the word cancer but it’s actually good news’: Discursive performativity of cancer and the identity of urological cancer services, Sociology of Health and Illness, 37(3), 340–354. 25847531 10.1111/1467-9566.12192

[bibr29-03063127231199217] Lancet. (2018). Immunotherapy: Hype and hope. The Lancet Oncology, 19(7), 845. 30084367 10.1016/S1470-2045(18)30317-6

[bibr30-03063127231199217] Lorimer J. (2016). Gut buddies: Multispecies studies and the microbiome. Environmental Humanities, 8(1), 57–76.

[bibr31-03063127231199217] Martin A. (2010). Microchimerism in the Mother(land): Blurring the borders of body and nation. Body & Society, 16(3), 23–50.

[bibr32-03063127231199217] Martin E. (1990) Toward an anthropology of immunology: The body as nation state. Medical Anthropology Quarterly, 4(4), 410–426.

[bibr33-03063127231199217] Martin E. (1994). Flexible bodies: Tracking immunity in American culture from the days of polio to the age of AIDS. Beacon Press.

[bibr34-03063127231199217] Matzinger P. (2002). The danger model: A renewed sense of self. Science, 296, 301–305. 11951032 10.1126/science.1071059

[bibr35-03063127231199217] Mol A. (2008). The logic of care: Health and the Problem of Patient Choice. Routledge.

[bibr36-03063127231199217] Mol A. Law J. (2004). Embodied action, enacted bodies: The example of hypoglycaemia. Body and Society, 10(2–3), 43–62.

[bibr37-03063127231199217] Murphy M. (2012). Seizing the means of reproduction: entanglements of feminism, health, and technoscience. Duke University Press. 10.1080/13648470.2015.1126081 28140612

[bibr38-03063127231199217] Napier D. A. (2002). The age of immunology: Conceiving a future in an alienating world. University of Chicago Press.

[bibr39-03063127231199217] Puig de La Bellacasa M. P. (2011). Matters of care in technoscience: Assembling neglected things. Social Studies of Science, 41(1), 85–106. 21553641 10.1177/0306312710380301

[bibr40-03063127231199217] Reisfield G. M. Wilson G. R. (2004). Use of metaphor in the discourse on cancer. Journal of Clinical Oncology, 22(19), 4024–4027. 15459229 10.1200/JCO.2004.03.136

[bibr41-03063127231199217] Rose N. (2007). The politics of life itself: Biomedicine, power, and subjectivity in the twenty-first century. Princeton University Press.

[bibr42-03063127231199217] Ross E. Swallow J. Kerr A. Cunningham-Burley S. (2019). Online accounts of gene expression profiling in early-stage breast cancer: Interpreting genomic testing for chemotherapy decision making, Health Expectations, 22(1), 74–82. 30387238 10.1111/hex.12832 PMC6351409

[bibr43-03063127231199217] Shildrick M. (2010). Some reflections on the socio-cultural and bioscientific limits of bodily integrity. Body & Society, 16(3), 11–22.

[bibr44-03063127231199217] Shildrick M. (2014). Visceral phenomenology: Organ transplantation, identity, and bioethics. In Zeiler K. Folkmars L. (Eds.), Feminist phenomenology and medicine (pp. 47–68). State University of New York Press.

[bibr45-03063127231199217] Sim D. (2018, October). Cutting edge’ CAR T cell immunotherapy approved in England. But is the NHS ready? Retrieved October 01, 2021, from https://scienceblog.cancerresearchuk.org/2018/10/05/cutting-edge-car-t-cell-immunotherapy-approved-in-england-but-is-the-nhs-ready/

[bibr46-03063127231199217] Sontag S. (1979). Illness as metaphor. Allen Lane.

[bibr47-03063127231199217] Stacey J. (1997) Teratologies: A cultural study of cancer. Routledge.

[bibr48-03063127231199217] Waldby C. Mitchell R. (2006). Tissue economies: Blood, organs, and cell lines in late capitalism. Duke University Press.

